# From urinary tract infection to deafness: community-acquired meningitis in an adult caused by hypervirulent *Klebsiella pneumoniae*—a case report

**DOI:** 10.1186/s12879-025-12340-0

**Published:** 2025-12-13

**Authors:** Xiaohui Yuan, Min Fang, Wei Lan, Xingqiang Zhou, Simin Yang, Tao Wang

**Affiliations:** 1 Department of General Practice, Huizhou Central People’s Hospital, Huizhou, Guangdong China; 2 Department of Neurology, Huizhou Central People’s Hospital, Huizhou, Guangdong China; 3 Department of Otolaryngology, Huizhou Central People’s Hospital, Huizhou, Guangdong China

**Keywords:** Hypervirulent *Klebsiella pneumoniae*, Meningitis, Urinary tract infection, Community-acquired, Deafness

## Abstract

**Background:**

Hypervirulent *Klebsiella pneumoniae* (hvKP) is an emerging pathogen capable of causing lethal metastatic infections in healthy individuals. While liver abscesses are well-described, the urinary tract is increasingly recognized as a primary portal for hematogenous dissemination.

**Case presentation:**

A 46-year-old man with previously undiagnosed diabetes mellitus presented with community-acquired urinary tract infection (CA-UTI) that rapidly progressed to bilateral sensorineural deafness and meningitis. Metagenomic next-generation sequencing (mNGS) of cerebrospinal fluid (CSF) identified *K. pneumoniae* harboring hypervirulence genes *rmpA* and *iucA*. Blood, urine, and CSF cultures yielded an ESBL-negative, pansensitive hvKP strain. Brain MRI revealed septic emboli, consistent with hematogenous dissemination.

**Conclusions:**

This case highlights the urinary tract as an underrecognized but lethal source of hvKP dissemination, particularly in diabetic patients. The rapid neurologic decline and permanent deafness highlight the need for early recognition and aggressive management. Virulence gene profiling should complement routine susceptibility testing. mNGS enabled rapid pathogen identification and guided targeted therapy. Clinicians must remain vigilant for CNS complications in diabetic patients with CA-UTI.

## Background

*Klebsiella pneumoniae* is a significant pathogen in both healthcare and community settings, characterized by two major concerns: antimicrobial resistance and hypervirulence [1]. While multidrug-resistant strains complicate treatment in hospitalized patients, the global spread of hypervirulent *K. pneumoniae* (hvKP) has become a global clinical concern due to its ability to cause invasive infections in otherwise healthy individuals [2, 3]. HvKP is defined by potent virulence mechanisms, and recent evidence indicates that the urinary tract serves as an important portal for disseminated infection [4, 5].

Community-acquired bacterial meningitis remains a neurological emergency with high morbidity and mortality [6]. Although *Streptococcus pneumoniae* and *Neisseria meningitidis* are the most common etiologies, Gram-negative pathogens such as *K. pneumoniae* are increasingly reported [7]. The progression from community-acquired urinary tract infection (CA-UTI) to meningitis is a rare but devastating manifestation of hvKP pathogenicity, often leading to delayed diagnosis and poor outcomes [8].

Despite growing awareness of the clinical significance of hvKP, detailed reports documenting its progression from CA-UTI to meningitis remain limited. This gap underscores the need for greater awareness and a better understanding of this severe disease progression. We present a case of an adult patient who developed the community-acquired meningitis originating from an hvKP CA-UTI.

## Case presentation

A 46-year-old male was admitted to Huizhou Central People’s Hospital on December, presenting with a 3-day history of high-grade fever and irritative voiding symptoms. The patient reported an abrupt onset of fever, with a peak temperature of 39 °C, accompanied by chills, urinary frequency, urgency, and difficulty voiding. There was no associated urinary pain, hematuria, back pain, nausea, or vomiting. His symptoms persisted without improvement, prompting him to seek medical attention. Notably, the patient reported a one-year history of recurrent polydipsia, polyuria, and xerostomia, which had not been medically evaluated. The patient reported no prior history of chronic diseases, infectious conditions, significant trauma, surgical procedures, or allergies. The patient had a history of smoking but quit one month before admission and reported that he abstained from alcohol and illicit drugs.

On admission, vital signs were as follows: temperature 38.1 °C, heart rate 99 bpm, respiratory rate 20 breaths/min, and blood pressure 137/83 mmHg. The patient was conscious and coherent. Physical examination revealed no neck stiffness or meningeal signs. Cardiopulmonary and abdominal examinations were unremarkable. Neurological examination showed normal muscle strength and tone in all four limbs, intact physiological reflexes, and negative pathological signs. Mild edema was noted in both lower extremities.

Initial laboratory tests revealed significant leukocytosis (15.47 × 10⁹/L) with a high neutrophil percentage (91.6%), markedly elevated inflammatory markers (C-reactive protein: 121.1 mg/L; procalcitonin: 4.16 ng/mL). The patient also presented with evidence of diabetic ketoacidosis (DKA), including hyperglycemia (random glucose: 16.58 mmol/L), metabolic acidosis (arterial pH: 7.27) with an elevated anion gap (16 mmol/L), and an increased β-hydroxybutyrate level (3.74 mmol/L). The hemoglobin A1c was 12.2%, consistent with longstanding hyperglycemia. Lactate level was 1.7 mmol/L. Liver function tests showed mild hyperbilirubinemia (total bilirubin: 38.21 µmol/L; direct bilirubin: 14.5 µmol/L) and hypoalbuminemia (30.90 g/L). Urinalysis indicated pyuria (27 white blood cells/µL) and microhematuria (8 red blood cells/µL). Serological tests for HIV, syphilis, and hepatitis B were negative. Renal function and coagulation profiles were within normal limits.

Imaging studies were consistent with an upper urinary tract infection. A renal ultrasound revealed perinephric stranding and a small right perinephric collection. An abdominal and pelvic computed tomography (CT) scan demonstrated bilateral renal swelling, thickening of the pelvicalyceal and ureteric walls, mild hydronephrosis, and bladder wall thickening.

Based on these findings, a diagnosis of severe urinary tract infection and new-onset diabetes with diabetic ketoacidosis was established. Initial management consisted of empirical antibiotic therapy with piperacillin-tazobactam (4.5 g intravenously every 8 h) and an intensive insulin regimen including preprandial aspart insulin (subcutaneously before breakfast, lunch, and dinner) and bedtime glargine insulin (subcutaneously at night), with subsequent titration based on blood glucose monitoring. Aggressive fluid resuscitation to correct ketosis and electrolyte replacement were also implemented.

On day 2, the patient’s condition deteriorated dramatically with the onset of bilateral hearing loss, gait instability, expressive dysphasia and psychomotor agitation. Otolaryngological consultation and pure-tone audiometry confirmed bilateral sensorineural deafness. An emergency non-contrast head CT showed a questionable hypodense area in the right occipital lobe. Although a cerebrovascular event was initially suspected by the neurology team, the constellation of severe infection and rapid neurological decline raised suspicion for a central nervous system (CNS) infection. Antibiotic therapy was escalated to meropenem (1 g every 8 h). Empiric intravenous methylprednisolone (80 mg qd) was administered for potential sudden sensorineural hearing loss after informed consent was obtained.

**Fig. 1:**
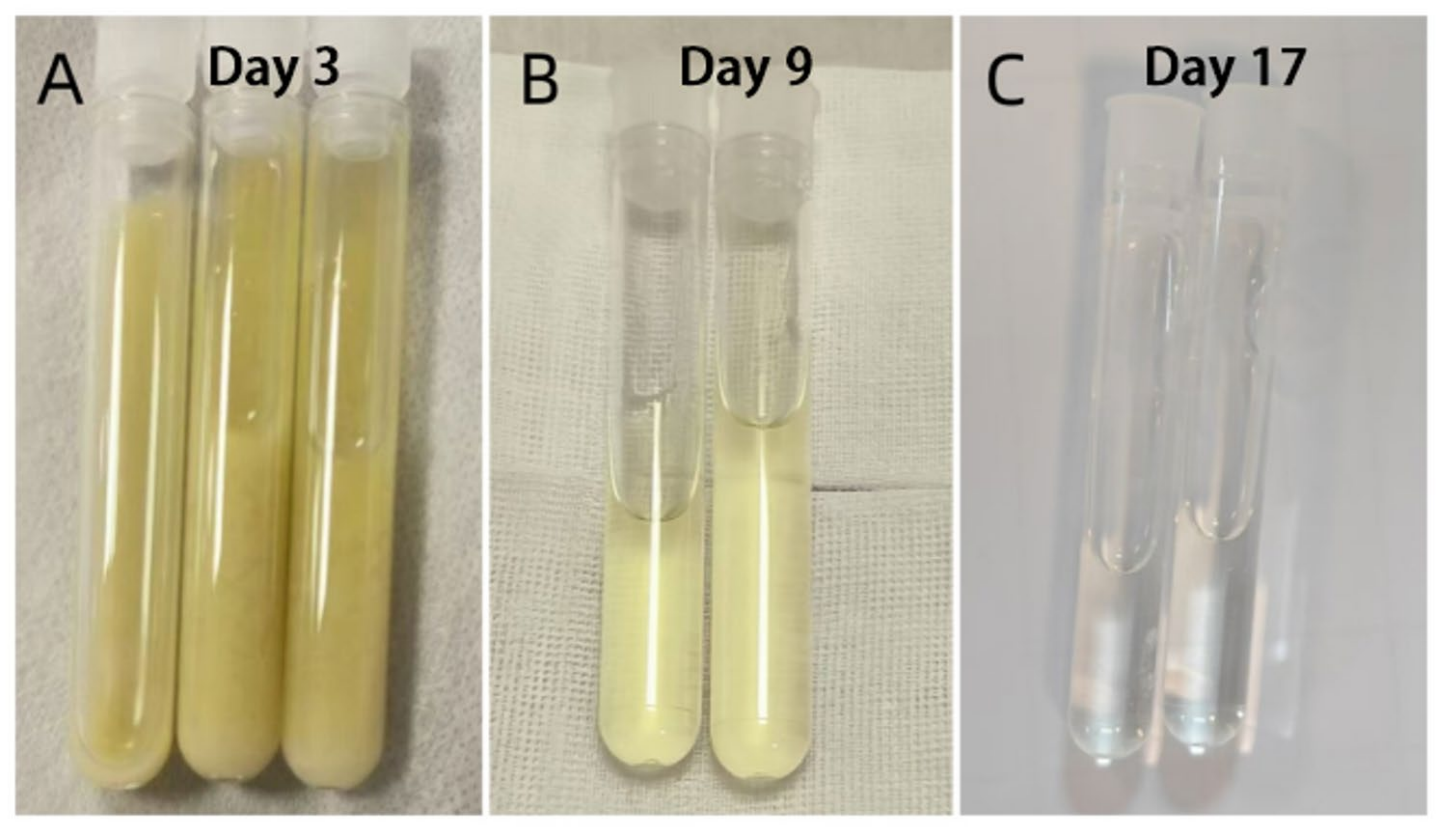
Changes in CSF appearance from turbid to clear. (**A**) on day 3; (**B**) on day 9; (**C**) on day 17

On day 3, the patient developed pronounced neck stiffness. A diagnostic lumbar puncture was immediately performed, revealing turbid, deep yellow cerebrospinal fluid (CSF) with an opening pressure exceeding the maximum measurable limit. CSF analysis confirmed purulent meningitis (Table 1; Fig. 1). CSF testing for cryptococcal antigen and *Mycobacterium tuberculosis* was negative. Meropenem was increased to a high-dose regimen (2 g every 8 h) targeting CNS penetration, and mannitol was administered to manage intracranial hypertension.

**Table 1:** CSF analysis results for the patient

CSF Data	On day 3	On day 9	On day17	On day 30
CSF appearance	Yellow and turbid	Light yellow	Light yellow	Transparent
CSF pressure (70–180 mm H_2_O)	>330 mm H_2_O	110	90	95
Protein (150–450 mg/L)	8668.4	3573.2	2081.6	1462.9
Chloride (120–132 mmol/L)	101.13	101.8	115.32	122.81
WBC (0–8 × 10^6^/L)	++++	9604	93	59
PMN cells (%)	/	65.4	18.3	1.7
Monocyte (%)	/	34.6	81.7	98.3
CSF glucose (2.22–3.89 mmol/L)	1.43	2.49	2.96	2.33
Blood glucose (<7.8 mmol/L)	19.1	7.3	5.8	4.1

*CSF* Cerebrospinal fluid, *WBC* white blood cell, *PMN* Polymorphonuclear Leukocyte, *++++* beyond the detection limit (> 40 white blood cells per high-power field), / not countable

**Fig. 2:**
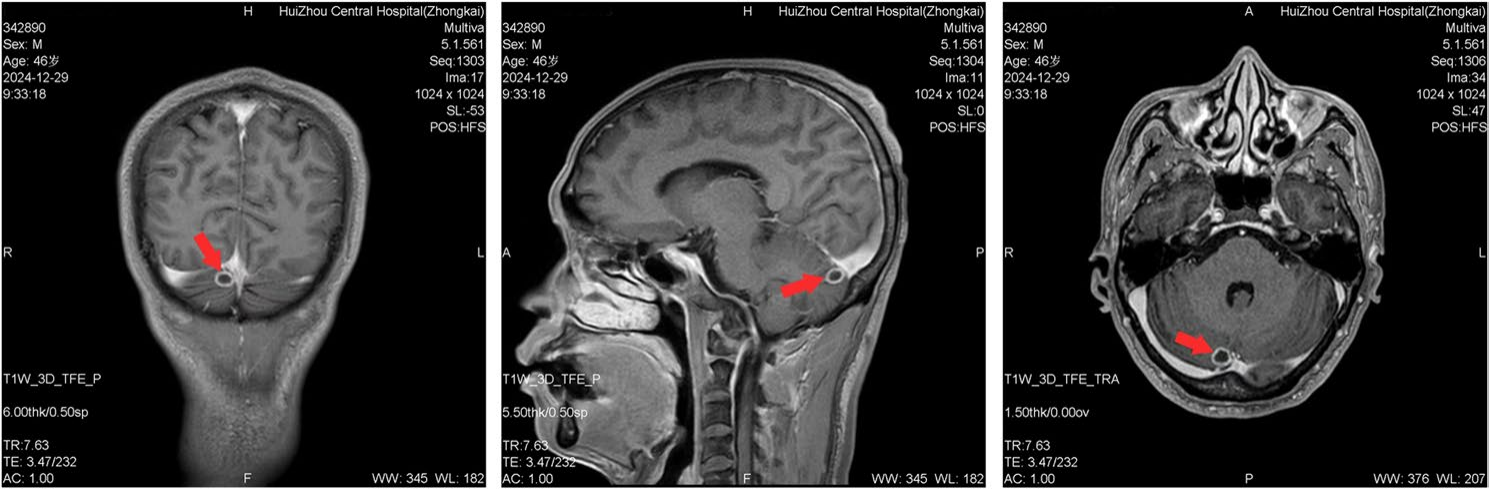
Brain MRI scans showed a 10-mm nodular lesion in the right cerebellar hemisphere (red arrow). *MRI* magnetic resonance imaging

On day 4, metagenomic next-generation sequencing (mNGS) of the CSF identified *Klebsiella pneumoniae* as the causative pathogen, with the presence of the hypervirulence-associated genes *rmpA* and *iucA* (aerobactin). No antimicrobial resistance genes were detected. Subsequent cultures of urine, blood, and CSF all grew ESBL-negative, string test-positive, fully susceptible to all tested antimicrobials *Klebsiella pneumoniae*. Antimicrobial susceptibility testing was performed using the broth microdilution method. Interpreted according to CLSI (or EUCAST) breakpoints, the strain was identified as a non-ESBL producer and was fully susceptible to all antimicrobials tested (Table 2). A CT scan of the chest and abdomen showed no other metastatic infectious foci. A follow-up brain MRI on day 13 showed nodular lesions in the right cerebellar hemisphere and left temporal lobe, suggestive of septic emboli or metastatic infection (Fig. 2).

**Table 2:** Antimicrobial susceptibility testing results for the *Klebsiella pneumoniae*

Antimicrobials	MIC (µg/mL)	Susceptibility	Antimicrobials	MIC (µg/mL)	Susceptibility
ESBL	Negative		Ceftazidime	≤ 0.12	Susceptible
Amoxicillin/ Clavulanic Acid	≤ 2	Susceptible	Cefepime	≤ 0.12	Susceptible
Piperacillin/ Tazobactam	≤ 4	Susceptible	Ertapenem	≤ 0.12	Susceptible
Cefoperazone/ Sulbactam	≤ 8	Susceptible	Trimethoprim/ Sulfamethoxazole	≤ 20	Susceptible
Cefuroxime	≤ 1	Susceptible	Amikacin	≤ 2	Susceptible
Cefuroxime Axetil	≤ 1	Susceptible	Levofloxacin	≤ 0.12	Susceptible
Cefoxitin	≤ 4	Susceptible	Tigecycline	≤ 0.5	Susceptible
Ceftriaxone	≤ 0.25	Susceptible			

The antimicrobial susceptibility test results of *Klebsiella pneumoniae* isolated from blood, midstream urine, and cerebrospinal fluid were all the same. Imipenem was susceptible​ (24‑mm inhibition zone by disk diffusion) against the isolate. *ESBL* Extended-Spectrum Beta-Lactamase, *MIC* Minimum Inhibitory Concentration

The patient’s clinical condition gradually improved with targeted antibiotic therapy. Fever and meningeal signs resolved, and inflammatory markers normalized. However, the bilateral profound deafness persisted, with repeat audiometric tests showing no recovery. On day 26, therapy was de-escalated to ceftazidime (2 g every 8 h) based on antimicrobial susceptibility.

After 32 days of hospitalization, the patient was discharged in a stable condition. He was prescribed a prolonged course of oral trimethoprim-sulfamethoxazole (160/800 mg twice daily, with a planned course of treatment of 1 weeks) and continued on insulin therapy. At 8-month follow-up, he remained free from recurrent systemic or neurological infections. Unfortunately, his bilateral deafness was permanent, and he declined further follow-up MRI imaging.

## Discussion

This case report describes a severe, community-acquired urinary tract infection (CA-UTI) caused by hypervirulent *Klebsiella pneumoniae* (hvKP) that rapidly progressed to meningitis and bilateral sensorineural deafness in a patient with previously undiagnosed diabetes. Our findings underscore the urinary tract as a critical yet underrecognized portal for hvKP dissemination, particularly in immunocompromised hosts such as diabetics, and highlight the pivotal role of advanced diagnostic tools like mNGS in enabling early pathogen identification and guiding targeted therapy.

The rapid progression from febrile UTI to meningitis and irreversible deafness within days illustrates the aggressive nature of hvKP. The identification of rmpA and iucA virulence genes via mNGS, along with the hypermucoviscous phenotype preliminarily determined by the string test, is consistent with established markers of hypervirulent Klebsiella pneumoniae (hvKP) associated with enhanced capsular polysaccharide production and siderophore-mediated iron acquisition [9, 10]. These characteristics contribute to systemic dissemination and invasion of the CNS. Studies have shown that the presence of the rmpA gene, an APACHE II score of ≥ 20, and the occurrence of septic shock are all important predictive factors for the development of septic metastatic lesions in *Klebsiella pneumoniae* infections [11]. The absence of ESBL production or antimicrobial resistance in this strain underscores that hypervirulence, not multidrug resistance, drives pathogenicity in such cases. This aligns with the reviews by Russo and Marr (2019), who noted that hvKP strains often remain pansensitive yet cause devastating clinical courses [2, 12, 13]. This distinction is critical, as hvKP infections often occur in community settings and affect healthy individuals [14] contrasting with healthcare-associated resistant strains. Therefore, in addition to routine susceptibility testing, virulence gene profiling is essential in cases of severe community-onset Klebsiella pneumoniae infections.

Notably, the patient’s undiagnosed type 2 diabetes mellitus (T2DM) likely contributed to disease severity. Hyperglycemia impairs neutrophil phagocytosis and capsular clearance, particularly for serotypes K1 and K2, which are common in hvKP. Diabetic ketoacidosis further exacerbates immune dysfunction, creating a permissive environment for hematogenous dissemination [10]. This case reinforces the emerging evidence that T2DM is a major risk factor for invasive hvKP infections, even in the absence of classical liver abscesses. When considering the treatment and prognosis of diabetic patients, repeated blood cultures are helpful for monitoring microbial clearance, understanding the impact of diabetes-related immune dysfunction on treatment efficacy, and timely adjusting the treatment plan to achieve effective microbial eradication [15].

This patient received a total of 32 days of inpatient intravenous antimicrobial therapy, followed by discharge and continuation of a 1-week oral course of trimethoprim/sulfamethoxazole. The decision for this extended treatment duration was based on the dynamic assessment of continuous clinical, laboratory, and imaging parameters. At the initial stage of treatment (days 2–3), empirical therapy was escalated and intensified to meropenem (2 g q8h) due to worsening neurological symptoms and confirmation of meningitis. Serial improvement in cerebrospinal fluid (CSF) parameters – including clearing of appearance from turbid to clear, reduction of white blood cells from “++++” to normal levels, and a significant decrease in protein – served as the core evidence for assessing the control of intracranial infection. The discovery of new intracerebral nodular lesions (septic emboli) on head MRI on day 13 directly demonstrated the ongoing risk of hematogenous dissemination, thereby supporting the necessity for prolonged, intensive intravenous therapy. After the resolution of clinical symptoms, normalization of inflammatory markers, and significant improvement in CSF parameters, therapy was de-escalated to ceftazidime on day 26 based on susceptibility results. This individualized management strategy, guided by clinical response and CSF parameters, aimed to complete an extended course to eradicate potential metastatic foci after infection control. This approach is entirely consistent with the management recommendations for disseminated infections caused by hvKP (particularly with CNS involvement) in the existing literature [16]. The literature recommends [17] that for such infections, intravenous therapy typically requires 4 to 6 weeks or longer, and discontinuation should be based on clinical improvement and normalization of CSF, rather than a predetermined fixed duration.

The patient’s sudden onset of bilateral hearing loss in this case represents a rare yet highly destructive complication of bacterial meningitis. The underlying mechanism may involve the dissemination of septic emboli to the cochlea or retrocochlear structures, as suggested by the presence of metastatic nodules in the cerebellum and temporal lobe observed on brain MRI. While such severe neurological sequelae have been reported in cases of hypervirulent hvKP meningitis [18], it is important to note that the incidence of hearing loss caused by K. pneumoniae is significantly lower compared to Streptococcus pneumoniae meningitis [19]. Streptococcus pneumoniae is widely recognized as the most common pathogen responsible for sensorineural hearing loss following meningitis, with an incidence as high as 30% or more [20, 21]. In contrast, meningitis caused by K. pneumoniae, particularly community-acquired hvKP strains, is relatively uncommon. The specific incidence of permanent hearing loss as a complication has not been clearly defined by large-scale epidemiological data. However, case reports suggest it is a severe and potentially underrecognized complication. The irreversible hearing loss observed in our patient underscores the necessity for heightened vigilance regarding potential central nervous system involvement in diabetic patients presenting with urinary tract infections, even in the absence of typical meningeal signs.

Compared to prior literature, this case adds to a limited but growing body of evidence linking CA-UTI to hvKP meningitis. In our patient, *Klebsiella pneumoniae* was isolated from blood, midstream urine, and cerebrospinal fluid cultures, with consistent antimicrobial susceptibility profiles. MRI findings-renal perinephric stranding, ureteric-wall thickening, and nodular cerebral lesions—suggest a haematogenous route of dissemination that began in the renal venous plexus, advanced through the vertebral plexus, and finally seeded the intracranial structures. While liver abscess remains the most documented source of hvKP dissemination [22], recent studies indicate that urinary tract infections are an underreported yet significant portal reported a series of hvKP UTIs progressing to metastatic infections [14], emphasizing the role of virulence genotyping in early diagnosis. Our case confirms these findings and extends them to include diabetic patients, a subgroup particularly vulnerable to atypical presentations.

The use of mNGS proved instrumental in rapidly identifying hvKP and its virulence genes, enabling timely escalation to carbapenem therapy [9]. Conventional cultures, though ultimately positive, required several days-a critical delay in a rapidly progressive infection. mNGS has emerged as a valuable tool in diagnosing central nervous system infections [23], especially when conventional methods are inconclusive or when rare pathogens are suspected. Our experience supports its integration into diagnostic algorithms for severe or atypical infections. Crucially, the reliability of mNGS in guiding therapy is underpinned by rigorous multi-layered quality control measures to mitigate contamination. These include the use of batch-specific negative controls to monitor background noise, bioinformatic subtraction based on laboratory-specific background databases, and integrated interpretation within the clinical context [24]. This technology enables rapid, unbiased pathogen identification, which directly impacts antimicrobial decision-making. When conventional methods yield negative results, mNGS can identify rare or fastidious pathogens, facilitating precise escalation of therapy [25]. More commonly, it allows for the rapid de-escalation from broad-spectrum empirical treatment to targeted narrow-spectrum therapy, or even supports the avoidance of unnecessary antibiotics by ruling out infection, thereby advancing antimicrobial stewardship [26].

However, this study also has certain limitations. Firstly, this is only a case report, and the extrapolation of the findings is limited. The complete pathological mechanism of hvKP causing meningitis and bilateral deafness through urinary tract origin, especially the exact pathway of hearing loss (such as cochlear embolism), is supported by imaging studies, but lacks final confirmation through more specialized inner ear imaging (such as high-resolution temporal bone CT or inner ear MRI). Secondly, although the existing treatment strategies have been successful, for hvKP central nervous system infections, especially those with disseminated lesions, there are no definite standards for the optimal total course of treatment, drug selection, and dosage based on large-scale clinical research. More case accumulation and prospective studies are still needed to reach a consensus.

## Conclusion

In summary, this case highlights the urinary tract as a lethal portal for hvKP dissemination, particularly in diabetics. Even when antimicrobial susceptibility is favorable, hvKP can disseminate from the urinary tract to the CNS within hours and cause permanent deafness.Clinicians should maintain a high index of suspicion for CNS involvement in diabetic patients with CA-UTI, even in the absence of classic signs. mNGS-directed early therapy is currently the best available tool for mitigating sequelae. Future research should focus on risk stratification, rapid diagnostic tools, and adjunctive therapies to minimise neurological sequelae.

## Data Availability

The datasets used and/or analyzed during the current study are available from the corresponding author on reasonable request.

## References

[1] Juan CH, Fang SY, Chou CH, Tsai TY, Lin YT. Clinical characteristics of patients with pneumonia caused by Klebsiella pneumoniae in Taiwan and prevalence of antimicrobial-resistant and hypervirulent strains: a retrospective study. Antimicrob Resist Infect Control. 2020;9(1):4. 31911832 10.1186/s13756-019-0660-x PMC6942382

[2] Russo TA, Marr CM. Hypervirulent Klebsiella pneumoniae. Clin Microbiol Rev. 2019;32(3):e00001–19. 31092506 10.1128/CMR.00001-19 PMC6589860

[3] Hu F, Pan Y, Li H, et al. Carbapenem-resistant Klebsiella pneumoniae capsular types, antibiotic resistance and virulence factors in china: a longitudinal, multi-centre study. Nat Microbiol. 2024;9(3):814–29. 38424289 10.1038/s41564-024-01612-1 PMC10914598

[4] Oh H, Heo ST, Kim M, Kang CH, Yoo JR. Devastating Community-Acquired bacterial meningitis caused by hypervirulent Klebsiella pneumoniae in an immunocompetent patient. J Clin Neurol. 2021;17(3):484–6. 34184461 10.3988/jcn.2021.17.3.484 PMC8242319

[5] Struve C, Roe CC, Stegger M, et al. Mapping the evolution of hypervirulent Klebsiella pneumoniae. mBio. 2015;6(4):e00630. 26199326 10.1128/mBio.00630-15 PMC4513082

[6] van de Beek D, Brouwer MC, Koedel U, Wall EC. Community-acquired bacterial meningitis. Lancet. 2021;398(10306):1171–83. 34303412 10.1016/S0140-6736(21)00883-7

[7] Abdeldaim GM, Strålin K, Korsgaard J, Blomberg J, Welinder-Olsson C, Herrmann B. Multiplex quantitative PCR for detection of lower respiratory tract infection and meningitis caused by *Streptococcus pneumoniae, Haemophilus influenzae and Neisseria meningitidis*. BMC Microbiol. 2010;10(3):310. 10.1186/1471-2180-10-310 PMC3016321 21129171

[8] Jin S, Xie H, Wang R. Otitis media progressing to Community-Acquired meningitis in diabetic patients: A case report of K2-ST375 hypervirulent Klebsiella pneumoniae and literature review. Infect Drug Resist. 2024;17:4707–16. 39494225 10.2147/IDR.S490828 PMC11529280

[9] Hetta HF, Alanazi FE, Ali MAS, et al. Hypervirulent Klebsiella pneumoniae: insights into Virulence, antibiotic Resistance, and fight strategies against a Superbug. Pharmaceuticals (Basel). 2025;18(5):724. 40430542 10.3390/ph18050724 PMC12115101

[10] Taha MS, Elkolaly RM, Elhendawy M, et al. Phenotypic and genotypic detection of hypervirulent Klebsiella pneumoniae isolated from Hospital-Acquired infections. Microorganisms. 2024;12(12):2469. 39770672 10.3390/microorganisms12122469 PMC11728040

[11] Lee SS, Chen YS, Tsai HC, et al. Predictors of septic metastatic infection and mortality among patients with Klebsiella pneumoniae liver abscess. Clin Infect Dis. 2008;47(5):642–50. 18643760 10.1086/590932

[12] Hassanin F, Khawjah D, Elkhamary S, Al Hussain H. Renal abscesses and endogenous endophthalmitis due to hypermucoviscous hypervirulent Klebsiella pneumoniae (HVKP). IDCases. 2021;24:e01130. 33996464 10.1016/j.idcr.2021.e01130 PMC8094904

[13] Huang S, Wei DD, Hong H, Chen S, Fan L-P, Huang Q-S, Du F-L, Xiang T-X, Li P, Zhang W, Wan L-G, Liu Y. Capture of mobile genetic elements following intercellular conjugation promotes the production of ST11-KL64 CR-hvKP. Microbiol Spectr. 2025;13(3):e0134724. 39898629 10.1128/spectrum.01347-24 PMC11878025

[14] Nagendra D, Chaudhuri S, Gupta N, et al. Prevalence, risk Factors, and clinical outcomes of hypervirulent Klebsiella pneumoniae strains among Klebsiella pneumoniae infections: A systematic review and Meta-analysis. Indian J Crit Care Med. 2025;29(4):370–93. 40322229 10.5005/jp-journals-10071-24957 PMC12045058

[15] Abdel Moneim A, Suleiman HA, Mahmoud B, Mabrouk D, Zaky MY, Mahmoud B. Viral clearance ameliorates hematological and inflammatory markers among diabetic patients infected with hepatitis C genotype 4. Clin Exp Med. 2020;20(2):231–40. 32076917 10.1007/s10238-019-00605-3

[16] Muñoz-Gómez S, Wirkowski E, Cunha BA. Post craniotomy extra-ventricular drain (EVD) associated nosocomial meningitis: CSF diagnostic criteria. Heart Lung. 2015;44(2):158–60. 25659927 10.1016/j.hrtlng.2015.01.003

[17] Rezar R, Jirak P, Lichtenauer M, Jung C, Lauten A, Hoppe UC, Wernly B. Partial oral antibiotic therapy is non-inferior to intravenous therapy in non-critically ill patients with infective endocarditis: review and meta-analysis. Wien Klin Wochenschr. 2020;132(23–24):762–9. 32040621 10.1007/s00508-020-01614-z PMC7732798

[18] Le Bourgeois F, Germanaud D, Bendavid M, Bonnefoy R, Desnous B, Beyler C, Blauwblomme T, Elmaleh M, Pierron C, Lorrot M, Bonacorsi S, Basmaci R. Kingella Kingae sequence type 25 causing endocarditis with multiple and severe cerebral complications. J Pediatr. 2016;169:326–e3261. 26651429 10.1016/j.jpeds.2015.10.091

[19] Jensen ES, Cayé-Thomasen P, Bodilsen J, et al. Hearing loss in bacterial meningitis Revisited-Evolution and recovery. Open Forum Infect Dis. 2023;10(3):ofad056. 36879624 10.1093/ofid/ofad056 PMC9985150

[20] Heckenberg SG, Brouwer MC, van der Ende A, Hensen EF, van de Beek D. Hearing loss in adults surviving Pneumococcal meningitis is associated with otitis and Pneumococcal serotype. Clin Microbiol Infect. 2012;18(9):849–55. 21958295 10.1111/j.1469-0691.2011.03668.x

[21] Sözen T, Bajin MD, Kara A, Sennaroğlu L. The effect of National Pneumococcal vaccination program on incidence of postmeningitis sensorineural hearing loss and current treatment modalities. J Int Adv Otol. 2018;14(3):443–6. 30541736 10.5152/iao.2018.6169 PMC6354544

[22] Lin JC, Siu LK, Fung CP, et al. Impaired phagocytosis of capsular serotypes K1 or K2 Klebsiella pneumoniae in type 2 diabetes mellitus patients with poor glycemic control. J Clin Endocrinol Metab. 2006;91(8):3084–7. 16720670 10.1210/jc.2005-2749

[23] Zhang X, Jiang C, Zhou C. Diagnosis of Enterococcus faecalis meningitis associated with long-term cerebrospinal fluid rhinorrhoea using metagenomics next-generation sequencing: a case report. BMC Infect Dis. 2021;21(1):1105. 34702199 10.1186/s12879-021-06797-y PMC8549229

[24] Zhang Z, Tian L. Validation of mNGS results using extensive lab and clinical data. BMC Microbiol. 2025;25(1):173. 40155846 10.1186/s12866-025-03908-6 PMC11951646

[25] Xie R, Shen J, Zhou L, Lu L, Zhi A, Sun D, Pei Y, Yu J, Zeng L, Gu G, Wang Y, Yu H, Chen Y, Ma X, Xie Z, Yang H. Rapid bacterial identification through multiplexed nucleic acid detection on a digital microfluidic platform for enhanced clinical intervention against infections. ACS Sens. 2025;10(4):2520–30. 39927898 10.1021/acssensors.4c02701

[26] Alba Fernandez J, Del Pozo JL, Leiva J, Fernandez-Alonso M, Aquerreta I, Aldaz A, Blanco A, Yuste JR. Impact of the acceptance of the recommendations made by a meropenem stewardship program in a university hospital: A pilot study. Antibiot (Basel). 2022;11(3):330. 10.3390/antibiotics11030330 PMC8944864 35326793

